# Acetylation of SAMHD1 at lysine 580 is crucial for blocking HIV-1 infection

**DOI:** 10.1128/mbio.01958-24

**Published:** 2024-08-20

**Authors:** Angel Bulnes-Ramos, Kerstin Schott, Jesse Rabinowitz, Charlotte Luchsinger, Cinzia Bertelli, Eri Miyagi, Corey H. Yu, Mirjana Persaud, Caitlin Shepard, Renate König, Baek Kim, Dmitri N. Ivanov, Klaus Strebel, Felipe Diaz-Griffero

**Affiliations:** 1 Department of Microbiology and Immunology, Albert Einstein College of Medicine, Bronx, New York, USA; 2 Host-Pathogen Interactions, Paul-Ehrlich-Institute, Langen, Germany; 3 Viral Biochemistry Section NIAID, NIH, Bethesda, Maryland, USA; 4 Department of Biochemistry, UTHSA, San Antonio, Texas, USA; 5 Department of Pediatrics, Center for ViroScience and Cure, School of Medicine, Emory University, Atlanta, Georgia, USA; 6 Children’s Healthcare of Atlanta, Atlanta, Georgia, USA; Rutgers-Robert Wood Johnson Medical School, Piscataway, New Jersey, USA

**Keywords:** HIV-1, SAMHD1, restriction, acetylation, K580, innate immunity, macrophages

## Abstract

In humans, sterile alpha motif (SAM) domain- and histidine–aspartic acid (HD) domain-containing protein 1 (SAMHD1) is a dNTPase enzyme that prevents HIV-1 infection in non-cycling cells, such as differentiated THP-1 cells and human primary macrophages. Although phosphorylation of threonine 592 (T592) in SAMHD1 is recognized as the primary regulator of the ability to prevent HIV-1 infection, the contributions of SAMHD1 acetylation to this ability remain unknown. Mass spectrometry analysis of SAMHD1 proteins derived from cycling and non-cycling THP-1 cells, primary cycling B cells, and primary macrophages revealed that SAMHD1 is preferentially acetylated at lysine residues 354, 494, and 580 (K354, K494, and K580). In non-cycling cells, SAMHD1 is preferentially acetylated at K580, suggesting that this post-translational modification may contribute to the ability of SAMHD1 to block HIV-1 infection. Consistent with this finding, we found that mutations in K580 disrupted the ability of SAMHD1 to block HIV-1 infection without affecting the ability of SAMHD1 to deplete cellular dNTP levels. Gene editing of SAMHD1 in macrophage-like cells revealed that an intact K580 is required for HIV-1 restriction. This finding suggests that K580 acetylation in SAMHD1 is essential for blocking HIV-1 infection. More importantly, we found that a larger proportion of SAMHD1 featuring K580 acetylation could be detected in human primary macrophages when compared to human primary monocytes. In agreement, we found that SAMHD1 is acetylated during the monocyte-to-macrophage differentiation process. This finding agrees with the idea that the blockade of HIV-1 infection in macrophages requires SAMHD1 acetylation.

**IMPORTANCE:**

The natural inhibitor of HIV-1, sterile alpha motif (SAM) domain- and histidine–aspartic acid (HD) domain-containing protein 1 (SAMHD1), plays a pivotal role in preventing HIV-1 infection of macrophages and dendritic cells, which are vital components of the immune system. This study unveils that SAMHD1 undergoes post-translational modifications, specifically acetylation at lysines 354, 494, and 580. Our research underscores the significance of these modifications, demonstrating that acetylation at residue K580 is indispensable for SAMHD1's efficacy in blocking HIV-1 infection. Notably, K580 is found in a critical regulatory domain of SAMHD1, highlighting acetylation as a novel layer of SAMHD1 regulation for HIV-1 restriction in humans. A comprehensive understanding of the regulation mechanisms governing this anti-HIV-1 protein is crucial for leveraging nature's defense mechanisms against HIV-1 and could pave the way for innovative therapeutic strategies.

## INTRODUCTION

The sterile alpha motif (SAM) domain- and histidine–aspartic acid (HD) domain-containing protein 1 (SAMHD1) potently restricts HIV-1 infection in non-cycling cells, such as macrophages, dendritic cells, and resting CD4^+^ T cells (1–9). The HIV-1 infection-blocking ability of SAMHD1 correlates with its dNTPase activity (10–12), which decreases dNTP levels in non-cycling cells. However, several known SAMHD1 mutations decrease cellular dNTP levels without restricting HIV-1 infection, suggesting that the dNTPase activity of SAMHD1 is necessary but not sufficient to enable the blockade of HIV-1 infection by SAMHD1 (13–18). Instead, the ability of SAMHD1 to block HIV-1 infection appears to be regulated by the phosphorylation status of the threonine residue at position 592 (T592) in SAMHD1 (13, 15, 17), with T592 phosphorylation inhibiting the ability of SAMHD1 to block HIV-1 infection (13, 15, 17). T592 dephosphorylation occurs upon the differentiation of a cell from the cycling to a non-cycling state, and multiple signals modulate T592 phosphorylation (15, 19–22).

As the name implies, SAMHD1 contains both a SAM domain and an HD domain. SAM domains are protein–protein interaction modules that mediate contact with other SAM domains (23) or non-SAM domain-containing proteins (23) and can also bind specific DNA sequences, acting as transcription activators or repressors (23). The HD domain is a dGTP-regulated dNTPase that decreases cellular dNTP levels (10–12, 24) and is required for SAMHD1 oligomerization and RNA-binding (18, 25–32). The HD domain has also been reported to have nuclease activity, but this activity remains under investigation (28, 33–36).

*In vitro* experiments by Lee et al. indicate that purified recombinant SAMHD1 protein serves as a substrate for the acetyltransferase arrest defect 1 (ARD1) (37), which catalyzes the *Nε-*lysine-acetylation of SAMHD1 on residue 405 (37). Separately, investigators found that sirtuin 1 deacetylates SAMHD1 at residue 354, which promotes DNA end resection by facilitating DNA binding at double-strand breaks (38). However, the effects of different SAMHD1 acetylation states on its ability to restrict HIV-1 infection have not yet been studied.

In this study, we examined endogenously expressed SAMHD1 in both cycling and non-cycling human cells and identified acetylation occurring at lysine residues 354, 494, and 580 (K354, K494, and K580). Restriction assays revealed that K580 acetylation is essential for the blockade of HIV-1 infectivity by SAMHD1 in human cells, whereas mutations targeting K580 did not affect the other known properties of SAMHD1, including nuclear localization, the ability to deplete cellular dNTP levels, and oligomerization. To test whether SAMHD1 K580 acetylation is altered by cellular differentiation from monocytes to macrophages, we developed a specific antibody targeting a SAMHD1 peptide with an acetylated K580 residue. Our results showed that a larger proportion of total SAMHD1 is acetylated in macrophages when compared to monocytes.

## RESULTS

### SAMHD1 is acetylated in human THP-1 cells

Previous *in vitro* experiments showed that pure recombinant SAMHD1 protein is *Nε-*lysine-acetylated at residue 405 by ARD1 (37). Acetylation regulates protein function by altering protein stability, subcellular localization, enzymatic activity, crosstalk with other post-translational modifications, and protein–protein or protein–DNA interactions (39) with effects on key cellular processes, including DNA damage repair, gene transcription, cell division, signal transduction, and autophagy (39). Therefore, we investigated whether SAMHD1 acetylation affects its ability to restrict HIV-1 infection. Because SAMHD1 only blocks HIV-1 infection in non-cycling cells, with no effect on cycling cells, we first determined the acetylation status of endogenously expressed SAMHD1 in both cycling and non-cycling cells.

**Fig 1:**
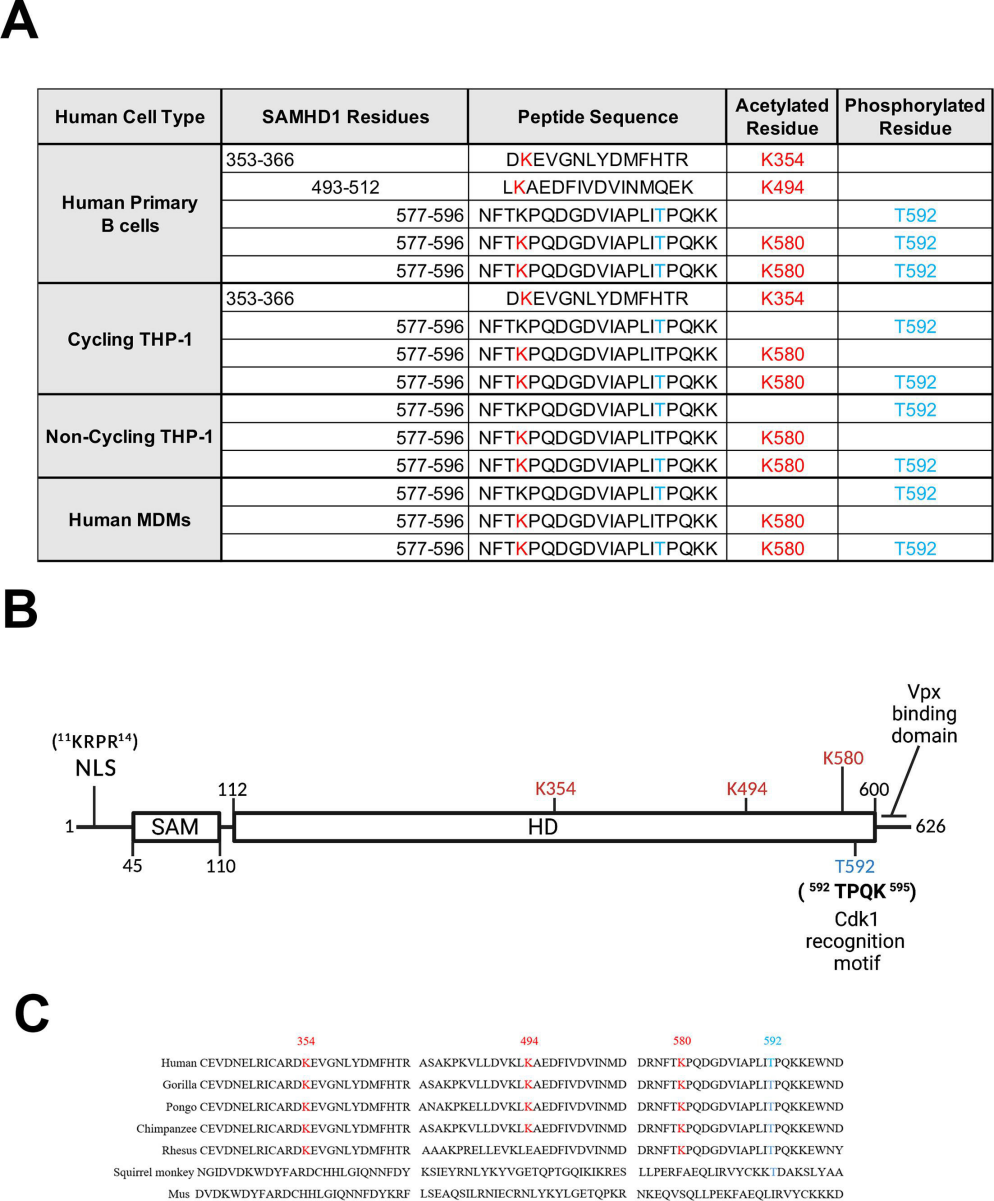
SAMHD1 acetylation in cycling and non-cycling human cells. (**A**) Mass spectrometry analysis of post-translational modifications associated with SAMHD1 proteins immunoprecipitated from human primary B cells, cycling THP-1 cells, non-cycling THP-1 cells, and human primary macrophages. SAMHD1-derived peptide sequences, with acetylated residues in blue and phosphorylated residues in red. Mass spectrometry analysis was performed at least four times, and highly reproducible modifications are shown. (**B**) The SAMHD1 protein, with the SAM and HD domains indicated. The nuclear localization signal (NLS), Cdk1 recognition motif, and Vpx-binding domain are illustrated. Acetylated lysine residues K354, K494, and K580 are shown in red. Phosphorylated threonine residue T592 is shown in blue. (**C**) The K354, K494, and K580 residues are well-conserved in SAMHD1 protein sequences across primates. MDM, monocyte-derived macrophage; SAMHD1, sterile alpha motif (SAM) domain- and histidine–aspartic acid (HD) domain-containing protein 1.

To determine which residues of endogenously expressed SAMHD1 are acetylated, we immunoprecipitated endogenously expressed SAMHD1 from different cell types, including cycling human primary B cells, cycling THP-1 cells, non-cycling THP-1 cells, and human primary macrophages. Immunoprecipitated proteins were separated by sodium dodecyl sulfate-polyacrylamide gel electrophoresis (SDS-PAGE) and analyzed by liquid chromatography–mass spectrometry to identify post-translational modifications. In cycling human primary B cells, endogenously expressed SAMHD1 is *Nε-*lysine-acetylated at residues K354, K494, and K580 (Fig. 1A), whereas in cycling THP-1 cells, SAMHD1 was only acetylated at residues K354 and K580 (Fig. 1A). Interestingly, in non-cycling THP-1 cells and macrophages, SAMHD1 was only acetylated at residue K580. Overall, our findings show that endogenously expressed SAMHD1 is *Nε-*lysine-acetylated at up to three different residues, K354, K494, and K580, in both cycling and non-cycling cells. In addition to identifying these acetylated residues, we also detected their non-acetylated counterparts (Fig. 1A and B). We assessed whether these acetylated residues are conserved across species by conducting sequence alignments. As shown in Fig. 1C, we observed that K354, K494, and K580 are well-conserved in SAMHD1 across primate species.

### Role of acetylation on the ability of SAMHD1 to block infection

**Fig 2:**
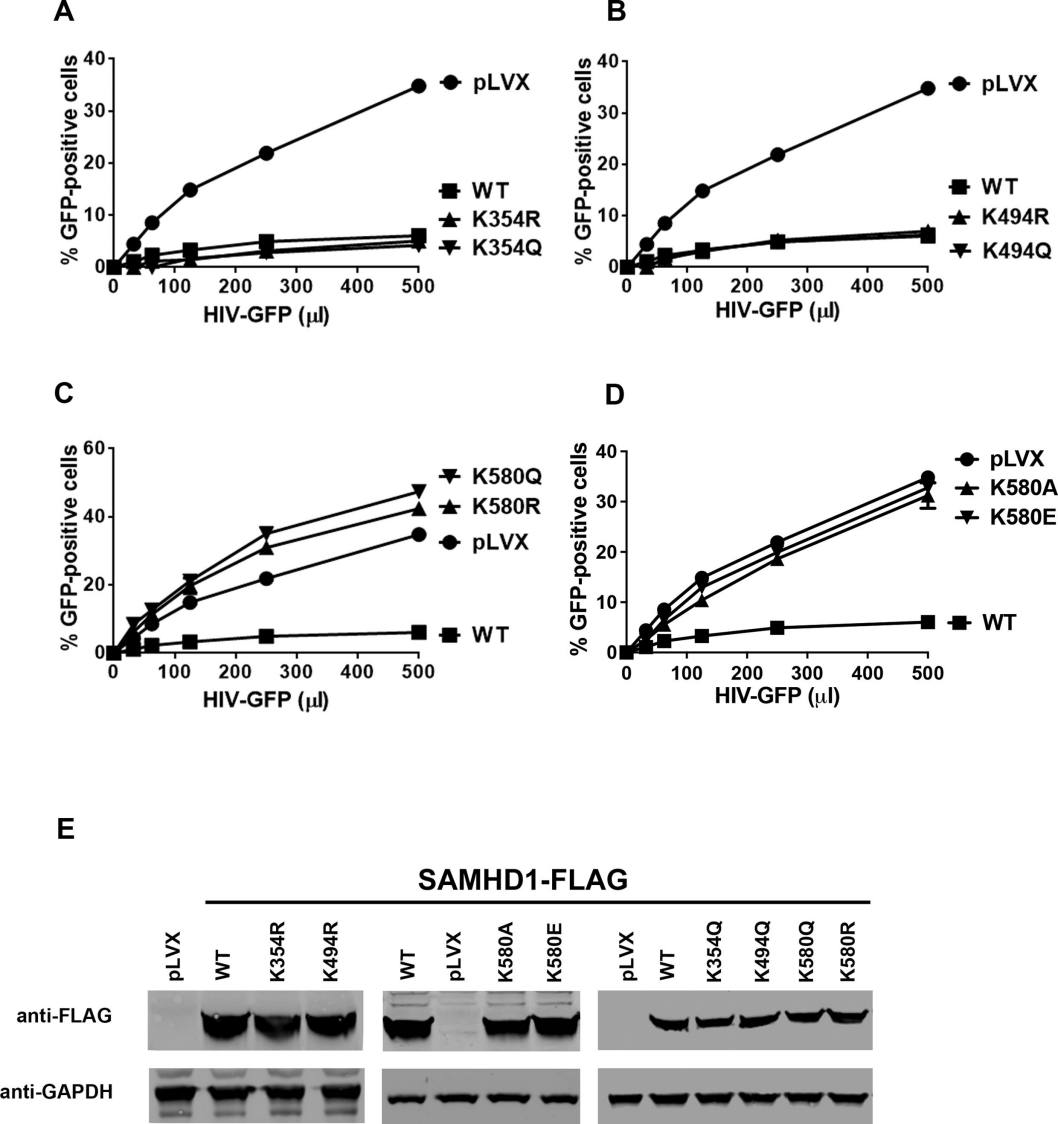
Mutations in acetylated K580 disrupt the ability of SAMHD1 to block HIV-1 infection. Phorbol 12-myristate 13-acetate (PMA)-treated, human monocytic U937 cells stably expressing FLAG-tagged wild-type (WT) or mutated SAMHD1 variants (A–D) were challenged with increasing amounts of HIV-1 virus expressing GFP as an infection. HIV-1-GFP challenges were also performed in PMA-treated U937 cells transduced with the empty vector, pLVX, as a control. After the infection challenge (48 h), cells were assessed by flow cytometry, and infectivity was determined as the percentage of GFP-positive cells. Experiments were performed three times, and a representative experiment is shown. (**E**) Western blotting using anti-FLAG antibodies was used to evaluate SAMHD1 expression in PMA-treated U937 cells expressing FLAG-tagged WT or mutated SAMHD1. GAPDH was used as a loading control. GAPDH, glyceraldehyde 3-phosphate.

Previous investigations have shown that post-translational modifications are important for the ability of SAMHD1 to block HIV-1 infectivity (13, 15, 16, 40), and we hypothesized that acetylation is important for this ability. We investigated the effects of acetylation on the ability of SAMHD1 to block HIV-1 infection in non-cycling U937 cells by first making SAMHD1 variants that cannot be acetylated. We generated human U937 cells that stably expressed these SAMHD1 variants and challenged these cells with increasing amounts of an HIV-1 construct that expresses green fluorescent protein (GFP) as an infection reporter (Fig. 2). As shown in Fig. 2A and E, replacing the K354 residue in SAMHD1 with either arginine (K354R) or glutamine (K354Q) residue had no effect on its ability to block HIV-1 infection. Similarly, mutation of K494 (K494R and K494Q) did not disrupt the HIV-1 restriction ability of SAMHD1 (Fig. 2B and E). However, as shown in Fig. 2C and E, the SAMHD1 variants K580R and K580Q, which prevent acetylation of K580, completely disrupted the ability of SAMHD1 to block HIV-1 infection. This finding is complemented by the finding that K580 is located on the surface of the protein structure (41). To further confirm our findings, we generated additional SAMHD1 variants, replacing K580 with alanine (K580A) or glutamic acid (K580E) residues. As shown in Fig. 2D and E, the SAMHD1 variants K580A and K580E were also unable to block HIV-1 infection, indicating that preventing acetylation at K580 disrupts the ability of SAMHD1 to block HIV-1 infection.

### Gene editing of SAMHD1 residue K580 in macrophage-like cells highlights the crucial role of K580 in the ability of SAMHD1 to inhibit HIV-1 infection

**Fig 3:**
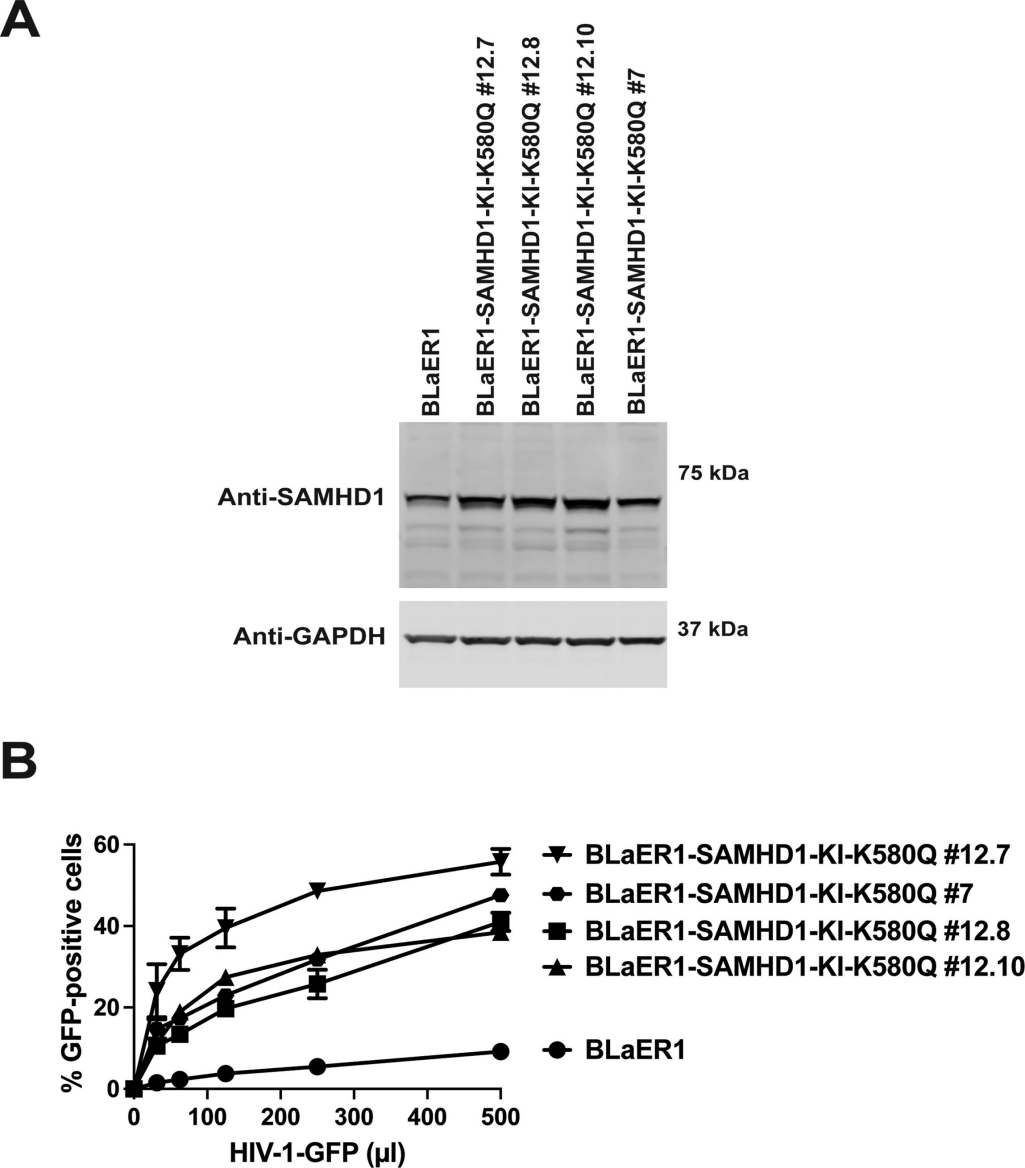
Expression of homozygous SAMHD1 K580Q loss HIV-1 restriction in transdifferentiated BLaER1 cells. Transdifferentiated BLaER1 clones endogenously expressing the SAMHD1 allele K580Q (BLaER1-SAMHD1-KI-K580Q) generated by CRISPR/Cas9 KI (**A**) were challenged with increasing amounts of HIV-1 viruses expressing GFP as a reporter of infection (HIV-1-GFP). Infectivity was determined 48 h post-infection by measuring the percentage of GFP-positive cells (**B**). Experiments were repeated three times, and a representative infection was shown.

The transdifferentiation of human BLaER1 cells to macrophage-like cells using 100 nM β-estradiol, 10 ng/mL of M-CSF, and 10 ng/mL of IL-3 results on cells that are resistant to HIV-1 infection, and this resistance is dependent upon the expression of SAMHD1 (42). To test the role of SAMHD1 K580 in restriction, we introduced the point mutation K580Q directly into the SAMHD1 gene locus by CRISPR/Cas9 knock-in (KI). To this end, we electroporated CRISPR/Cas9 ribonucleoproteins together with a single-stranded DNA correction template by electroporation of BLaER1 cells followed by an allele-specific PCR (KASP-genomic assay screening). Rigorous validation by Sanger sequencing and quantitative genomic PCR to exclude large genomic deletions was performed. We identified single-cell clones that were homozygous for the mutation K580Q in the SAMHD1 locus of BLaER1 cells (BLaER1-SAMHD1-KI-K580Q). As shown in Fig. 3A, all homozygous clones for the SAMHD1-K580Q allele expressed similar levels of SAMHD1 when compared to wild-type (WT) BLaER1 cells. In agreement with our U937 cells expressing the different SAMHD1 variants for residue K580, we found that transdifferentiated BLaER1 cells expressing SAMHD1-K580Q lose the ability to block HIV-1 infection when compared to WT BLaER1 cells. These experiments showed an intact K580 residue in endogenously expressed SAMHD1 is important for the restriction of HIV-1.

### Effects of acetylation on SAMHD1 subcellular localization

**Fig 4:**
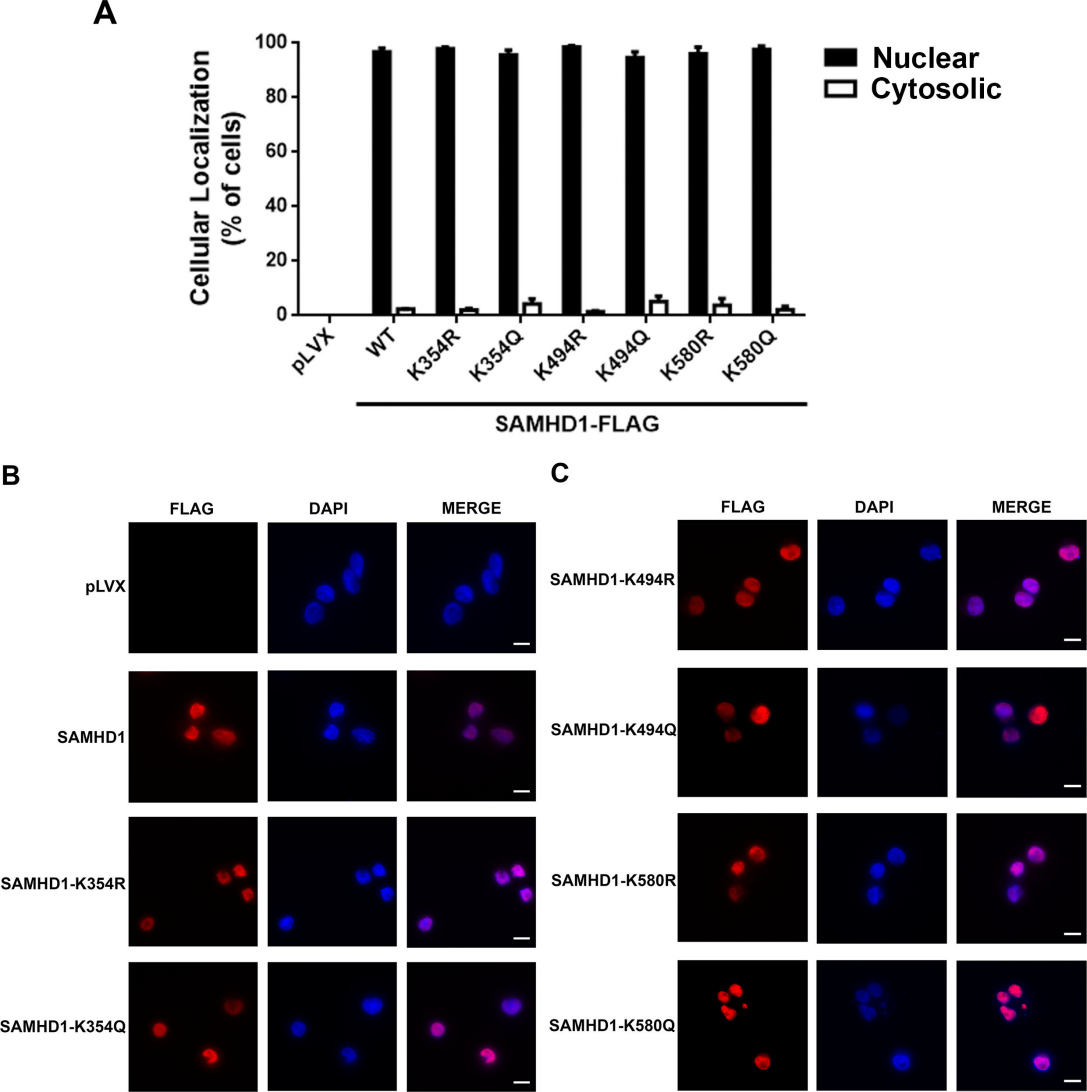
Intracellular distribution of WT and mutated SAMHD1 protein. (A) The nuclear and cytoplasmic distributions of WT and mutated SAMHD1 proteins in PMA-treated U937 cells were quantified using a manual scoring system. The mean and standard deviation for three independent experiments are shown. In every experiment, 200 cells were counted. (B and C) PMA-treated U937 cells expressing the indicated SAMHD1-FLAG variants were fixed and stained using antibodies against FLAG (red). Cellular nuclei were stained with DAPI (blue). The scale bar is 10 µm. DAPI, 4′,6-diamidino-2-phenylindole.

Because protein acetylation is known to alter subcellular localization (39), we explored whether acetylation affects the nuclear localization of SAMHD1 using SAMHD1 variants in non-cycling U937 cells. We generated non-cycling U937 cells stably expressing FLAG-tagged SAMHD1 variants and used anti-FLAG antibodies in immunofluorescence assays to visualize the subcellular localization of SAMHD1 (Fig. 4). We visually quantified 100 cells in each of three independent experiments, which revealed that all of the SAMHD1 variants displayed nuclear localization similar to WT SAMHD1 (Fig. 4A through C). These experiments indicate that disrupting K580 acetylation has no effect on the nuclear localization of SAMHD1.

### Effect of acetylation on the ability of SAMHD1 to deplete the cellular dNTP levels

**Fig 5:**
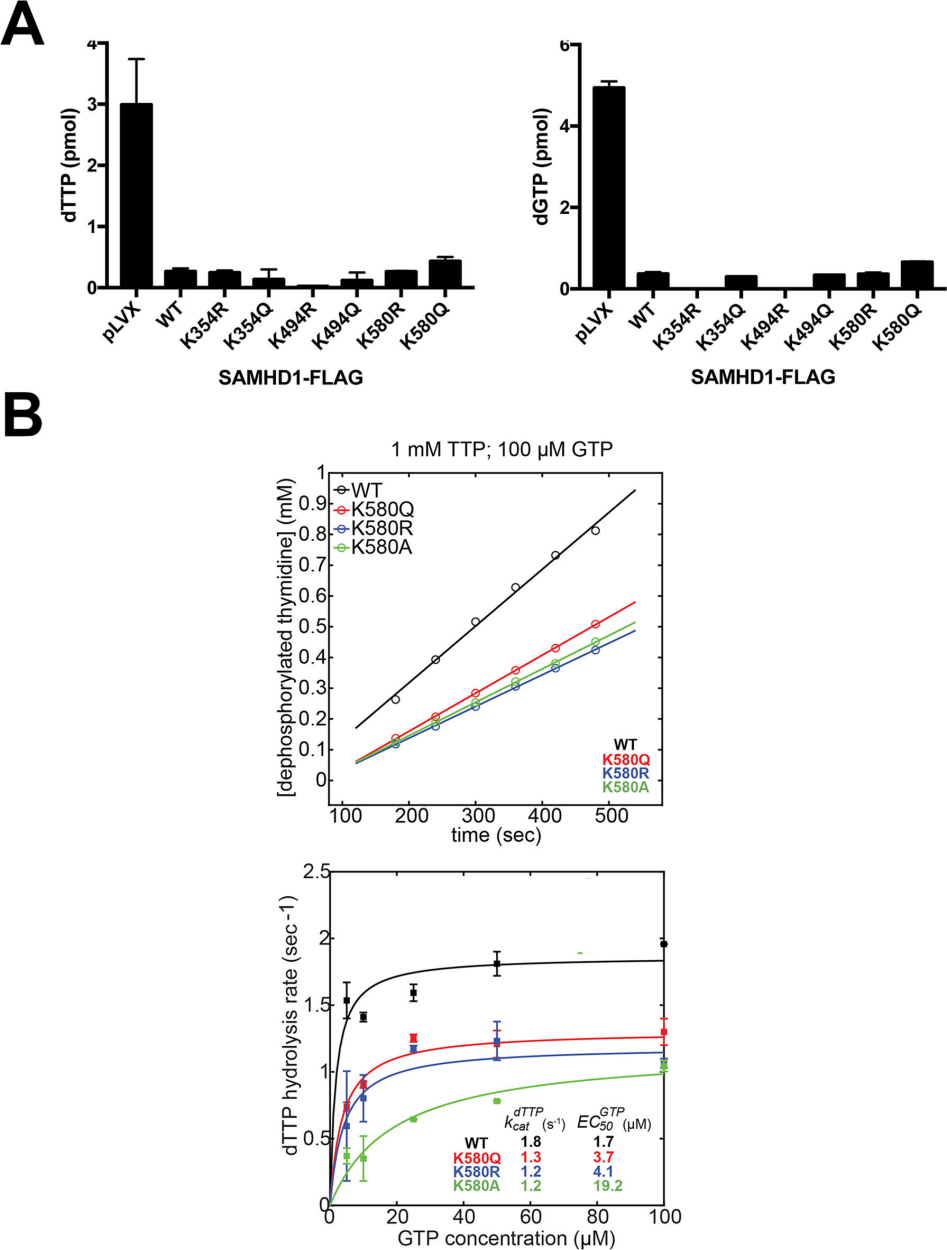
Acetylation variants affect the ability of SAMHD1 to alter cellular dNTP levels. (**A**) Total dGTP and dTTP levels were measured in PMA-treated human monocytic U937 cells stably expressing WT or mutated SAMHD1 protein using a primer extension assay. The mean and standard deviation from three independent experiments are shown. (**B**) SAMHD1 dNTP hydrolysis. The dNTPase activity of purified recombinant SAMHD1 variants was measured by monitoring dTTP hydrolysis over time (upper panel). Reactions were performed using 1 mM of dTTP and hydrolysis rates were determined from the steady-state segments of the progress curves (lower and upper panels). EC_50_^GTP^ and *k*_cat_ parameters were determined by analyzing the dependency of the dTTP hydrolysis rate on GTP concentration (lower panel). Experiments were repeated three times, and representative experiments are shown.

The ability of SAMHD1 to deplete cellular dNTP levels in non-cycling cells is one of its best-studied functions. To evaluate whether disrupting acetylation affects the ability of SAMHD1 to deplete cellular dNTP levels, we measured cellular dNTP levels in non-cycling human U937 cells stably expressing SAMHD1 variants. As shown in Fig. 5A, cellular dNTP levels remained unaffected, regardless of the SAMHD1 variant expressed, indicating that SAMHD1 acetylation does not contribute to the dNTP depletion activity of SAMHD1.

To further investigate whether SAMHD1 variants were defective in the dNTPase activity, we tested the enzymatic activity of purified recombinant SAMHD1_114-626_ variants (Fig. 5B). dNTPase activity was measured by monitoring the intensity of the characteristic nuclear magnetic resonance (NMR) signal of the product resulting from dTTP hydrolysis, which is the measurement of dephosphorylated thymidine over time. Reactions were performed using 1 mM of dTTP substrate, and hydrolysis rates were determined from the steady-state segments of the progress curves shown in Fig. 5B, upper panel. To study whether the enzymatic activity is affected, we determine the EC_50_^GTP^ (half-maximal effective concentration) and *k*_cat_ (first-order rate constant that determines the reaction rate when the enzyme is saturated by substrate) parameters for each of the shown SAMHD1 variants by analyzing the dependency of dTTP hydrolysis rate on GTP concentration (Fig. 5B, lower panel). We observed that the SAMHD1_114-626_ K580 variants displayed a reduced steady-state dTTP hydrolysis rate compared to WT protein, but that the effect was modest corresponding to less than twofold reduction in the *k*_cat_ parameter for the mutant variants (Fig. 5B). This modest reduction in *k*_cat_ agrees with the ability of the SAMHD1 mutants to reduce cellular dNTP concentrations as efficiently as the WT protein. In addition, we measured the dependence of the steady-state dTTP hydrolysis rate on GTP concentration as a way to monitor the allosteric activation properties of SAMHD1 (Fig. 5B). The effect of K580 mutations on the EC_50_^GTP^ parameter was more pronounced than the reduction of *k*_cat_ values, but all apparent EC_50_^GTP^ were still significantly lower than the physiological GTP concentrations, once again in agreement with the ability of the mutant SAMHD1 variants to efficiently deplete cellular dNTP pools.

### Effect of acetylation on SAMHD1 oligomerization

**Fig 6:**
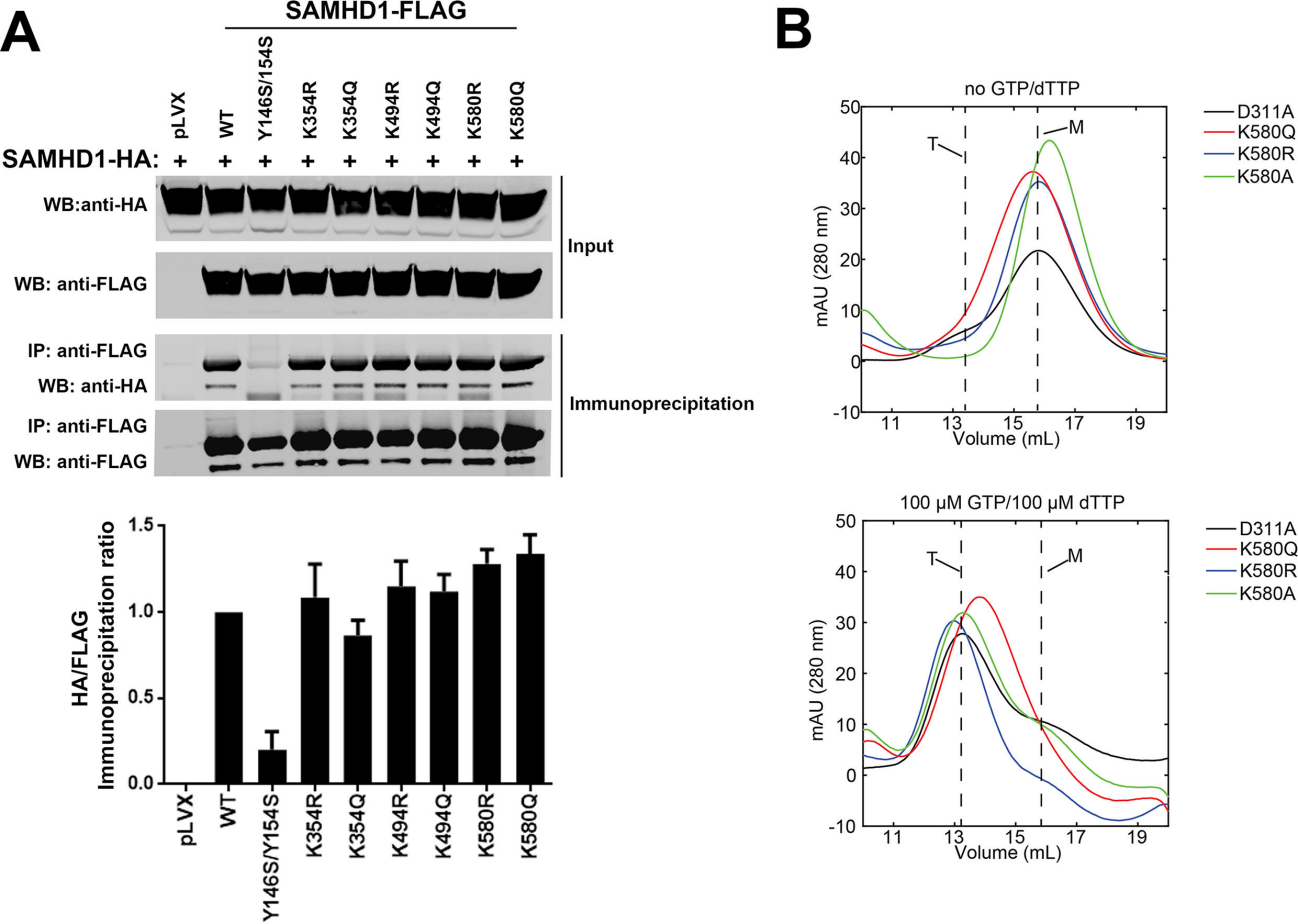
Oligomerization capability of SAMHD1 acetylation variants. (**A**) Human 293T cells were co-transfected with a plasmid expressing HA-tagged WT SAMHD1 and a plasmid expressing FLAG-tagged WT or mutated SAMHD1. Cells were lysed 24 h after transfection and analyzed by western blotting using anti-HA and anti-FLAG antibodies (input). Lysates were immunoprecipitated using anti-FLAG agarose beads, and FLAG peptide was used to elute immunoprecipitated proteins from beads. Western blotting was performed using anti-HA and anti-FLAG antibodies (immunoprecipitation). As a control, we utilized the SAMHD1 variant Y146S/154S, which is oligomerization defective. Similar results were obtained in three independent experiments, and representative data are shown. WB, western blot; IP, immunoprecipitation. (**B**) Size exclusion chromatography of SAMHD1 variants. Purified recombinant SAMHD_114-626_ variants were separated on a Superdex G200 column in the presence (lower panel) or absence (upper panel) of nucleotide triphosphates to determine their oligomerization state. The retention volumes for the monomeric and tetrameric forms are indicated. Experiments were repeated three times and representative figures are shown.

*In vitro* experiments using purified SAMHD1 demonstrate that dNTPase activity requires tetramerization, which is supported by data from us and others showing that SAMHD1 exists in an oligomeric form in various cell lines (27, 29, 30, 32, 41). We tested whether SAMHD1 variants that prevent acetylation have any effect on oligomerization. As shown in Fig. 6A, all of the tested SAMHD1 variants were able to oligomerize, whereas our control, SAMHD1-Y146S/Y154S, which has previously been shown to disrupt oligomerization, failed to oligomerize (29). These results indicate that the acetylation status of SAMHD1 does not regulate oligomerization.

To further investigate the oligomerization state of SAMHD1 variants, we monitored dNTP-dependent SAMHD1 oligomerization by size exclusion chromatography. As shown in Fig. 6B (upper panel), in the absence of dNTPs purified SAMHD_114-626_ proteins containing the variants K580Q, K580R, and K580A are predominantly in monomeric state (M), as evidenced by their retention volume of approximately 15.8–16.2 mL on the Superdex G200 column. As a control, we utilized the SAMHD_114-626_ variant D311A, which does not have enzymatic activity. By contrast, the addition of 100 µM GTP and 100 µM dTTP resulted in a significant change in the retention volume of all SAMHD1 variants (Fig. 6B, lower panel). These results showed that all variants tetramerize (T) in the presence of nucleotide triphosphates. As a control, we utilized the SAMHD_114-626_ variant D311A, which does not have enzymatic activity; therefore, it will not hydrolyze dNTPs in the size exclusion buffer (Fig. 6A and B). These results showed that SAMHD1 mutations on K580 do not affect the tetramerization ability of the protein.

### SAMHD1 acetylation in human THP-1 cells and primary macrophages

**Fig 7:**
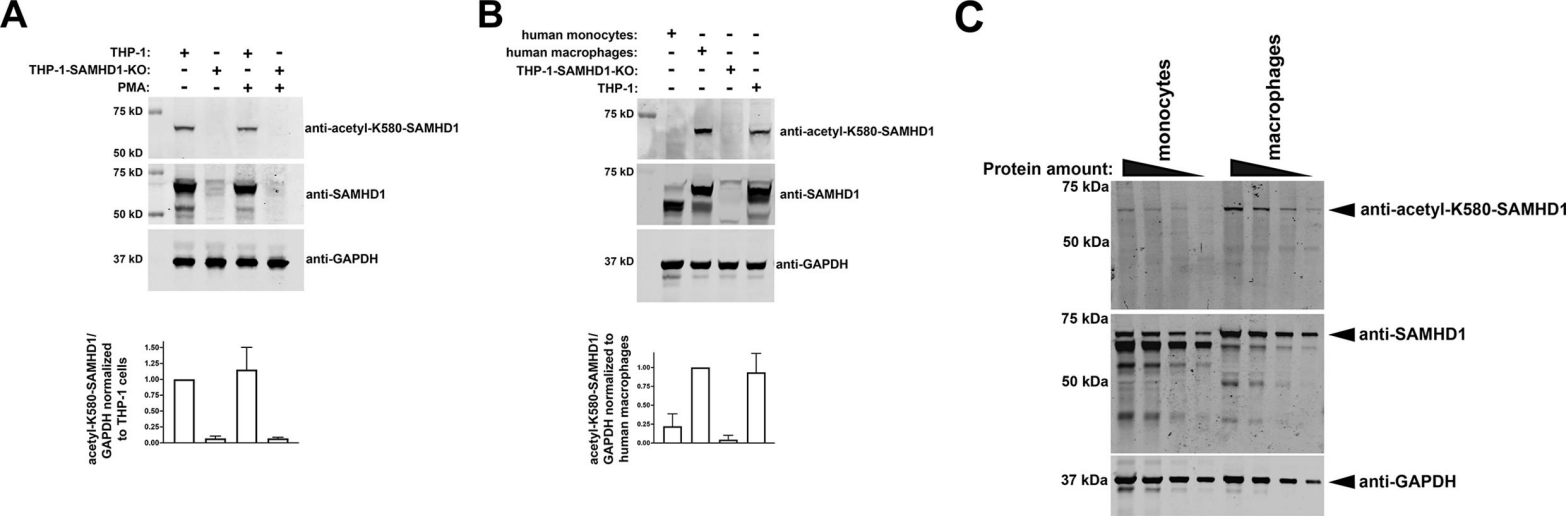
Human primary macrophages, but not monocytes, exhibit SAMHD1 acetylation at K580. (**A**) PMA-treated WT and SAMHD1 knockout (SAMHD1-KO) THP-1 cells were tested for acetylation at SAMHD1 K580 by western blotting using a specific antibody against a SAMHD1 peptide featuring acetylation at K580 (anti-acetyl-K580-SAMHD1). Total SAMHD1 levels were assessed to control for SAMHD1 expression (anti-SAMHD1). GAPDH was used as the loading control. (**B**) Similarly, human primary monocytes and macrophages were tested for acetylation at SAMHD1 K580, controlling for total SAMHD1 expression (anti-SAMHD1), with GAPDH as the loading control. As a control, we used WT and SAMHD1 knockout (SAMHD1-KO) THP-1 cells. (**C**) Increasing amounts of cellular extracts derived from human primary monocytes and macrophages were analyzed for acetylation at SAMHD1 K580, controlling for total SAMHD1 expression (anti-SAMHD1), with GAPDH as the loading control.

The ability of SAMHD1 to block HIV-1 infection is limited to non-cycling cells, a hallmark of this function, and this ability is prevented by the phosphorylation of T592. Extensive investigations have revealed that SAMHD1 is phosphorylated at T592 in cycling cells, whereas dephosphorylation occurs when cells enter a non-cycling stage. Our investigations revealed the acetylation of K580 in endogenously expressed SAMHD1 and showed that preventing acetylation via K580 point mutations abolished the ability of SAMHD1 to block HIV-1 infection without affecting other known properties of SAMHD1. We measured the levels of SAMHD1 acetylation in both cycling and non-cycling cells to determine whether the K580 acetylation status differs between these stages. We developed a rabbit polyclonal antibody against a SAMHD1-derived peptide that contains a *Nε-*acetylated K580 residue (anti-acetyl-K580-SAMHD1). We initially evaluated SAMHD1 acetylation in cycling and non-cycling THP-1 cells (Fig. 7A), which display similar levels of SAMHD1 acetylation. To control for antibody specificity, we performed similar experiments using THP-1 cells that are knockout for SAMHD1 (THP-1-SAMHD1-KO). We analyzed all samples for total SAMHD1 levels and used glyceraldehyde 3-phosphate dehydrogenase (GAPDH) as a loading control (Fig. 7A). Our initial assessment indicates no difference in acetylation status between cycling and non-cycling cells in human cell lines.

We then investigated K580 acetylation levels in human primary macrophages. As shown in Fig. 6B, SAMHD1 is acetylated at K580 in macrophages, demonstrating that this modification also occurs in human primary cells. As controls, we also measured K580 acetylation in cycling and non-cycling THP-1 cells and analyzed all samples for total SAMHD1 and GAPDH levels. Our experiments revealed that monocytes preferentially express a smaller version of SAMHD1 (Fig. 7B), which likely represents either a degradation product or a splicing variant. However, these experiments revealed that full-length SAMHD1 protein is poorly expressed in monocytes. We assessed the K580 acetylation levels of full-length SAMHD1, normalized against total SAMHD1 expression levels, comparing between monocytes and macrophages (Fig. 7C), which revealed that a larger proportion of SAMHD1 was acetylated in macrophages when compared to monocytes. Overall, these experiments revealed that monocytes express a smaller SAMHD1 variant, although whether this represents a splicing variant or a degradation product has not yet been determined, and that a larger proportion of SAMHD1 is acetylated in macrophages than in monocytes.

**Fig 8:**
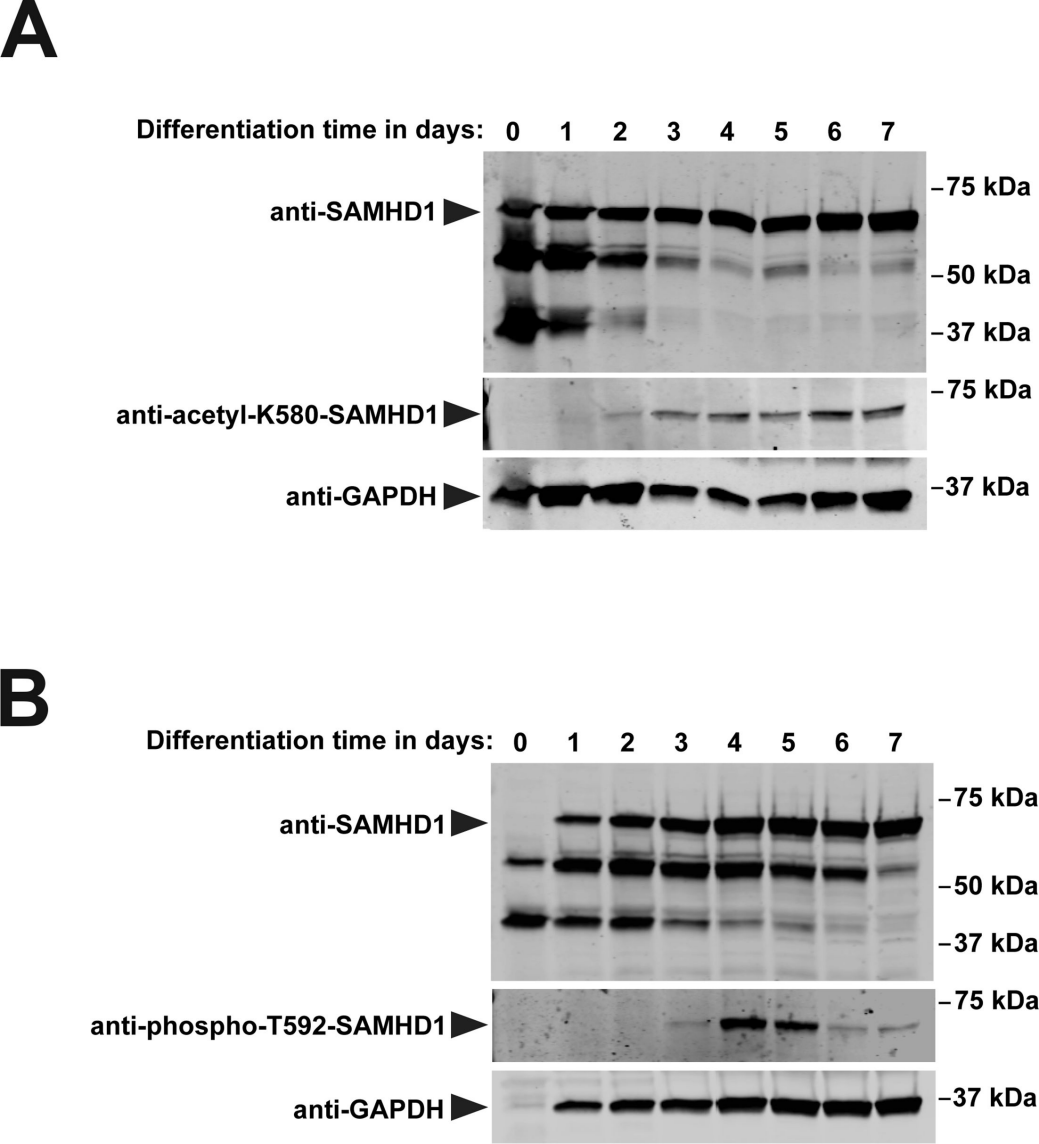
Changes in SAMHD1 acetylation and phosphorylation during monocyte to macrophage differentiation. Human primary monocytes were differentiated into macrophages, with total protein extracts collected daily for 7 days. These extracts were analyzed by western blot using anti-acetyl-K580-SAMHD1 (A) and anti-phospho-T592-SAMHD1 (B) antibodies. Additionally, all samples were probed using an anti-total-SAMHD1 antibody (anti-SAMHD1). An anti-GAPDH antibody was used as a loading control for all samples.

To investigate how acetylation of residue K580 changes during the differentiation of monocytes into macrophages, we conducted a time course experiment and measured K580 acetylation (Fig. 8A) and T592 phosphorylation (Fig. 8B) daily over 7 days. As shown in Fig. 8A, the fraction of SAMHD1 acetylated at residue K580 increases over time as monocytes differentiate into macrophages. Concurrently, the smaller fragments of SAMHD1 decrease over time, suggesting that SAMHD1 is less degraded or that its splicing variants change during differentiation (Fig. 8B). Interestingly, the fraction of SAMHD1 phosphorylated at residue T592 peaks between days 4 and 5 and becomes completely unphosphorylated when monocytes are fully differentiated (Fig. 8B).

## DISCUSSION

Protein acetylation regulates various cellular processes (39). The present study identified three different *Nε-*lysine-acetylated residues in endogenously expressed SAMHD1: K354, K494, and K580. All three residues were acetylated in cycling human primary B cells, but only K354 and K580 were acetylated in cycling THP-1 cells. Interestingly, non-cycling THP-1 cells and macrophages were only acetylated at K580, suggesting that the differential acetylation status of K580 regulates SAMHD1 function. Although several residues surrounding K580 are under positive selection (43), this particular lysine does not show the feature of positively selected residues during evolution (43).

SAMHD1 acetylation at K580 was also found to be important for its ability to block HIV-1 infection, as our mutagenesis studies revealed that replacing K580 with R, Q, A, or E residues inhibited this ability. These experiments suggested that an intact residue K580 is required for restriction. In agreement, gene editing of K580 in the human BLaER1 cell line showed that an endogenously expressed SAMHD1 bearing the K580Q change was unable to block HIV-1 infection demonstrating that mutations in K580 are detrimental to the ability of SAMHD1 to block HIV-1 infection.

SAMHD1 K580 point mutations did not affect the ability of SAMHD1 to deplete dNTP levels in non-cycling cells. SAMHD1 K580 point mutations join a growing number of SAMHD1 mutations, including T592D/E, H376A, Q548A, and A525T, that are able to reduce dNTP levels but lose their ability to prevent HIV-1 infection (16, 18). These variants may all affect some undiscovered SAMHD1 function or regulatory mechanism that is essential for HIV-1 restriction. Unlike point mutations targeting K580, the point mutations we tested targeting K354 and K494 did not appear to have any effects on the ability of SAMHD1 to block HIV-1 infection.

Previous investigations revealed that phosphorylation at T592 governs both the ability of SAMHD1 to prevent HIV-1 infection and the flexibility of the SAMHD1 C-terminal domain, particularly those residues that follow the HD domain (16, 44). C-terminal domain flexibility is important for the ability of SAMHD1 to interact with nucleic acids (18, 27, 45), and the acetylation of K580 may be necessary for the formation of structural determinants that are involved in these interactions (18, 27, 45). A close look at the SAMHD1 structure shows that the side chains for K580, D585, and T592 share a confined space, suggesting that K580 acetylation and T592 phosphorylation may regulate the conformation of this region, which ultimately regulates the ability of SAMHD1 to block HIV-1 infection. Further supporting the idea that SAMHD1 acetylation at K580 is important for HIV-1 restriction, histone deacetylase inhibitors possess SAMHD1-dependent anti-HIV-1 activity in macrophages (46), which indirectly suggests that SAMHD1 acetylation is important for restriction.

Our investigations revealed that full-length SAMHD1 is acetylated at K580 in human primary macrophages, whereas a much smaller proportion of SAMHD1 is acetylated at K580 in human primary monocytes, which are the cellular precursors of macrophages. Interestingly, in macrophages, the full-length SAMHD1 protein appears to be the most commonly detected isoform, whereas in human monocytes, the most commonly detected SAMHD1 isoform is a smaller fragment (~60 kDa). Monocytes may express a SAMHD1 splicing variant lacking exons 8 and 9 (∆8–9), resulting in the loss of dNTPase activity (deletion of residues 285–354) (47). However, no differences in the sizes of SAMHD1 protein were observed in cycling and non-cycling THP-1 cells, nor were any differences in the K580 acetylation status detected. Alternatively, SAMHD1 K580 acetylation may protect the full-length protein from degradation in macrophages, as has been described for other proteins (48, 49), leading to full-length SAMHD1 being the predominant isoform expressed in macrophages (47). Unlike in human primary monocytes, full-length SAMHD1 is acetylated in human monocytic THP-1 cells, indicating that cycling THP-1 cells and human primary monocytes may display differences in SAMHD1 expression and modification.

Our work shows that SAMHD1 K580 acetylation is important for HIV-1 restriction activity, and future investigations remain necessary to clarify the role played by K580 acetylation in the biology of SAMHD1.

## MATERIALS AND METHODS

### Cell lines and plasmids

Human THP-1 (American Type Culture Collection [ATCC]) and U937 (ATCC #CRL 1593) and BLaER1 (Sigma, SCC165) cells were grown in RPMI supplemented with 10% fetal bovine serum and penicillin/streptomycin. Plasmids expressing the codon-optimized SAMHD1 sequence fused to either an HA (pLVX-SAMHD1-HA) or FLAG (pLVX-SAMHD1-FLAG) epitope have been previously described (27).

### Generation of U937 cells stably expressing SAMHD1 variants

Lentiviral vectors encoding WT or mutated SAMHD1 sequences fused to a FLAG epitope were created using the pLVX vector (Clontech). Recombinant viruses were produced in HEK293T cells by co-transfecting the pLVX plasmids with a lentiviral packaging mix containing the vesicular stomatitis virus G (VSV-G) envelope glycoprotein, which allows for efficient entry into a wide range of vertebrate cells (50). Transduced human monocytic U937 cells were selected in 0.4 µg/mL puromycin (Sigma).

### Protein analysis

Cellular proteins were extracted with radioimmunoprecipitation assay, as previously described (51). Western blotting was performed using anti-FLAG (Sigma), anti-GAPDH (Sigma), or anti-HA (Sigma) antibodies to detect proteins of interest. Secondary antibodies, conjugated to Alexa Fluor 680, against rabbit and mouse were obtained from Li-Cor. Bands were detected by scanning blots using the Li-Cor Odyssey Imaging System in the 700 nm channel.

### Infection with retroviruses expressing the GFP

Recombinant retroviruses expressing GFP and pseudotyped with the VSV-G glycoprotein were prepared as described (52). For infections, phorbol-12-myristate-3-acetate (PMA)-treated cells (6 × 10^4^) were seeded in 24-well plates for 16 h. PMA was used at a final concentration of 10 ng/mL. Subsequently, cells were incubated with the indicated retrovirus for 48 h at 37°C. The percentage of GFP-positive cells was determined by flow cytometry (BD Celesta). Viral stocks were titrated by serial dilution on human A549 cells.

### SAMHD1 oligomerization assay

Approximately 1.0 × 10^7^ human HEK293T cells (Invitrogen) were co-transfected with plasmids encoding FLAG-tagged and HA-tagged mutated or WT SAMHD1. After 24 h, cells were lysed in 0.5 mL whole-cell extract (WCE) buffer (50 mM Tris [pH 8.0], 280 mM NaCl, 0.5% IGEPAL, 10% glycerol, 5 mM MgCl_2_, 50 µg/mL ethidium bromide, 50 U/mL benzonase tail [Roche]). Lysates were centrifuged at 14,000 rpm for 1 h at 4°C. After centrifugation, lysates were pre-cleared using protein A-agarose (Sigma) for 1 h at 4°C. A small aliquot of each pre-cleared lysate was stored as Input. Pre-cleared lysates containing the tagged proteins were incubated with anti-FLAG-agarose beads (Sigma) for 2 h at 4°C. Anti-FLAG-agarose beads were washed three times in WCE buffer, and immune complexes were eluted using 200 µg FLAG tripeptide/mL in WCE buffer. The eluted samples were separated by SDS-PAGE and analyzed by western blotting using either anti-HA or anti-FLAG antibodies.

### Indirect immunofluorescence microscopy and subcellular localization

PMA-treated U937 cells stably expressing WT or mutated SAMHD1 were plated on 24-mm-diameter cover glasses for 18 h. Subsequently, cells were fixed, permeabilized, and stained using anti-FLAG antibodies (Sigma-Aldrich, F7425) at a dilution of 1:1,000, as previously described (53). To fluorescently visualize the proteins, we utilized anti-rabbit Cy2-conjugated antibodies. Before mounting, nuclei were stained with 4′,6-diamidino-2-phenylindole (DAPI) as previously described (53). For each sample, 200 cells were visually inspected to determine nuclear or cytosolic localization.

### Immunoprecipitation assay

Human primary B cells, cycling or non-cycling THP-1 cells (as described previously), and human monocyte-derived macrophages (5–10 × 10^6^ cells) were cultured in RPMI supplemented with 10% fetal bovine serum and penicillin/streptomycin. Cells were pelleted and lysed by the addition of 1 mL protein lysis buffer (50 mM Tris HCl [pH 8.0], 280 mM NaCl, 0.5% IGEPAL, 10% glycerol, 5 mM MgCl_2_, proteases cocktail inhibitors) supplemented with benzonase (50 U/mL) and ethidium bromide (50 µg/mL). After 1 h of agitation at 4°C, samples were centrifuged at 14,000 rpm for 1 h at 4°C. Pre-cleared cell lysates were incubated for 1 h with agarose beads coupled with rabbit anti-SAMHD1 antibodies. Following extensive washes in lysis buffer, bound proteins were eluted by boiling in Laemmli buffer for 10 min. Samples were separated by SDS-PAGE, and protein bands (~75 kDa) were extracted and stored at −20°C. Mutations and post-translational modifications of SAMHD1 were analyzed by mass spectrometry in the Gigi Lab (Harvard Medical School).

### Determination of cellular dNTP levels

For each cell type, 2–3 × 10^6^ cells were used to determine cellular dNTP levels. Cells were washed twice with 1× phosphate-buffered saline, pelleted, and resuspended in ice-cold 65% methanol in Millipore-grade water. Samples were vortexed for 2 min and incubated at 95°C for 3 min. Subsequently, samples were centrifuged at 14,000 rpm for 3 min, and the supernatant was transferred to a new tube for complete drying and methanol removal using a speed vac. Dried samples were resuspended in Millipore-grade water. An 18-nucleotide primer labeled at the 5′ end with ^32^P (50-GTCCCTGTTCGGGCGCCA-30) was annealed at a 1:2 ratio with each of four separate 19-nucleotide templates (50-NTGGCGCCCGAACAGGGAC-30), where “N” represents nucleotide variations at the 5′ end. Reaction conditions were as follows: 200 fmoles of template primer, 2 µL of 0.5 mM dNTP mix (positive control) or dNTP cell extract, 4 µL of excess HIV-1 RT, 25 mM Tris HCl (pH 8.0), 2 mM dithiothreitol, 100 mM KCl, 5 mM MgCl_2_, and 10 µM oligo(dT) in a final volume of 20 µL. The reaction was incubated at 37°C for 5 min before being quenched with10 µL of 40 mM EDTA and 99% (vol/vol) formamide at 95°C for 5 min. The extended primer products were resolved on a 14% urea-PAGE gel and analyzed using a phosphoimager. The extended products were quantified using QuantityOne software to quantify the percent volume of saturation. The quantified dNTP content of each sample was accounted for based on its dilution factor, adjusting each sample volume to obtain a signal within the linear range of the assay.

### CRISPR/Cas9-mediated KI of the SAMHD1 gene in BLaER1 cells

Specific K580 mutations were introduced in the SAMHD1 locus of BLaER1 cells as described previously (42). In brief, 200 pmol crRNA targeting SAMHD1 exon 15 (5′-CAGAAATTTCACCAAGCCGC-3′, IDT) was mixed with 200 pmol tracrRNA (Dharmacon) and incubated for 30 min at 37°C. crRNA:tracrRNA duplexes were incubated with 40 pmol Cas9-NLS (QB3 Macrolab) to form Cas9 ribonucleoproteins (RNPs) (54). BLaER1 cells (1 × 10^6^) were nucleofected with RNPs and the respective Alt-R HDR Donor Oligos (100 pmol, IDT) using the 4D-Nucleofector X Unit (program: DN-100) and SF Cell Line Kit (Lonza). ssDNA HDR donor templates contained additional silent mutations in order to perform PCR-based screening for successful KI in single cell-derived BLaER1 clones (K580Q: 5′AATTGTGCAAAGTTTGTGAGTAACAGGCCACCTACCTGGGGCTGGGTGAAATTTCGGTCTGCACACCACTGAACAAAATATTGT-3′; K580R: 5′-AATTGTGCAAAGTTTGTGAGTAACAGGCCACCTACCTGCGGCCGGGTGAAGTTTCTGTCTGCACACCACTGAACAAAATATTGT-3′). After incubation at 32°C for 48 h in RPMI medium supplemented with Alt-R Enhancer V2 (1:500, IDT), single cell-derived clones of nucleofected BLaER1 cells were generated by limited dilution. Upon confluency, cells were (partially) harvested for DNA preparation by resuspension of cells in 50 µL lysis buffer (0.2 mg/mL proteinase K, 1 mM CaCl_2_, 3 mM MgCl_2_, 1 mM EDTA, 1% Triton X-100, 10 mM Tris [pH 7.5]) and heating (10 min at 65°C + 15 min at 95°C).

To identify potential KIs in single cell-derived BLaER1 clones, PCR was carried out with GoTaq G2 DNA polymerase (Promega) using K580- or mutation-specific primers (K580_for: 5′-CAGAAATTTCACCAAGCCGCAG-3′; K580Q_for: 5′-CCGAAATTTCACCCAGCCCC-3′; K580R_for: 5′-CAGAAACTTCACCCGGCCGC-3′). All PCRs were performed with the same reverse primer (K580_rev: 5′-GCTAACTAATATACTGAAGTAACGAGGTTTAGAAAC-3′). The following PCR conditions were used: initial denaturation at 95°C for 2 min, followed by 35 cycles of denaturation at 95°C for 30 s, annealing at 62°C for 30 s, extension at 72°C for 35 s, and a final extension at 72°C for 5 min. PCR products were separated using 1.5% (wt/vol) agarose gels. Homozygous KIs were further confirmed by Sanger sequencing after PCR amplification using primers encompassing the K580 site in SAMHD1 (KI_K580_TIDE_for: 5′-ggaatagttaggagcctagggacc-3′; KI_K580_TIDE_rev: 5′-GCTAACTAATATACTGAAGTAACGAGGTTTAG-3′) and KAPA HiFi Hot Start ReadyMix (Roche). In order to exclude large DNA deletions in the SAMHD1 locus, the presence of both alleles was assessed with quantitative genomic PCR using SAMHD1-specific PrimeTime Mini qPCR Assay (IDT; for 5′-TCACAACTTCTGCCAGAGAAA-3′, probe: 5′-/56-FAM/TTTGCAGAGCAGCTGATTCGAGT/3BHQ_1/-3′; rev: 5′-TCTTGCGGCATACAAACTCT-3′), human TERT TaqMan Copy Number Reference Assay (ThermoFisher) and PrimeTime Gene Expression Master Mix (IDT) on a CXF384 cycler (BioRad).

### NMR-monitored dNTPase assay

Measurements of SAMHD1-catalyzed dNTP hydrolysis were performed by monitoring intensities of characteristic NMR signals of dNTP substrates and dN products of the reaction as previously described (32). Briefly, NMR samples containing 1 mM dTTP, varying concentrations of GTP, and 1 µM of WT or mutant SAMHD1114-626 constructs were prepared in the buffer containing 50 mM Tris, pH 7.5, 150 mM NaCl, 5 mM MgCl_2_, 5 mM DTT, and 10% D_2_O. 1H NMR spectra were acquired at regular time intervals and the relative peak intensity of the H6 proton signal of deoxythymidine triphosphate (substrate) versus deoxythymidine nucleoside (product) was measured as a function of time. The rate of dNTP hydrolysis was determined by linear fitting of the hydrolysis reaction curves using MATLAB software (Mathworks).

### Studies of SAMHD1114-626 tetramerization by size exclusion chromatography

SAMHD1114-626 tetramerization was investigated by size exclusion chromatography on a Superdex 200 10/300 Gl column (Cytiva; GE Life Sciences) and an AKTA Purifier chromatography system (Cytiva, GE Life Sciences) (18). Samples containing WT and mutant SAMHD1114-626 constructs were injected onto the size exclusion chromatography (SEC) column either without nucleotide triphosphates or in the presence of 100 µM dTTP and 100 µM GTP included both in the injected sample and the SEC running buffer (50 mM Tris, pH 8, 100 mM NaCl, 5 mM MgCl_2_, and 5 mM DTT). Retention volumes for different SAMHD1 variants at different conditions were determined by continuously monitoring the 280 nm absorbance of the column eluate. NMR samples of oligonucleotides with or without SAMHD1 present were prepared in the buffer containing 50 mM Tris, pH 7.5, 150 mM NaCl, 5 mM MgCl_2_, and 10% D_2_O. 31P NMR spectra were acquired on a Bruker 700-MHz spectrometer equipped with a 5 mm room-temperature broadband RF probe. 1H NMR spectra were acquired on a 500-MHz spectrometer equipped with a 1.7 mm cryoprobe.

Kinetics of dNTP hydrolysis catalyzed by SAMHD1 was investigated using an NMR-based assay. Proton NMR spectra were recorded at regular time intervals and the relative peak intensity of the H6 proton signal of deoxythymidine triphosphate (substrate) versus deoxythymidine nucleoside (product) was measured as a function of time. The rate of dNTP hydrolysis was determined by linear fitting of the hydrolysis reaction curves using MATLAB software (Mathworks).
